# An unusual presentation of propylthiouracil-induced anti-MPO and PR3 positive ANCA vasculitis with associated anti-GBM antibodies, IgA nephropathy and an IgG4 interstitial infiltrate: a case report

**DOI:** 10.1186/s12882-020-01964-w

**Published:** 2020-07-23

**Authors:** J. R. Galante, C. P. Daruwalla, I. S. D. Roberts, R. Haynes, B. C. Storey, M. J. Bottomley

**Affiliations:** 1 Oxford Kidney Unit, Churchill Hospital, Oxford University Hospitals NHS Foundation Trust, Oxford, OX3 7LE UK; 2 Department of Cellular Pathology, John Radcliffe Hospital, Oxford University Hospitals NHS Foundation Trust, Oxford, OX3 9DU UK; 3 MRC Population Health Research Unit, Clinical Trial Service Unit & Epidemiological Studies Unit, Nuffield Department of Population Health, Richard Doll Building, Old Road Campus, Roosevelt Drive, Headington, Oxford, OX3 7LF UK

**Keywords:** Case report, NCA-associated vasculitis, Propylthiouracil, Anti-GBM disease, IgA nephropathy, IgG4-related disease

## Abstract

**Background:**

A number of disease processes can culminate in rapidly progressive glomerulonephritis, including pauci-immune focal segmental necrotising glomerulonephritis, usually seen with positive serum antineutrophil cytoplasmic antibodies (ANCA). Propylthiouracil (PTU) has been associated with drug-induced ANCA-associated vasculitis (AAV), with antibodies against myeloperoxidase (MPO) and proteinase 3 (PR3) present individually and together having been recognised. ‘Double-positive’ vasculitis with ANCA and anti-glomerular basement membrane (GBM) antibodies has also been reported in association with PTU treatment. We present a case of PTU-induced anti-MPO and PR3 positive ANCA vasculitis with associated anti-GBM antibodies, IgA nephropathy and an IgG4 interstitial infiltrate.

**Case presentation:**

A 51-year-old man presented 2 weeks after re-commencing propylthiouracil (PTU) treatment for Graves’ disease, with a severe acute kidney injury and haemato-proteinuria. He demonstrated positive titres for autoantibodies to PR3 (76.9 IU/mL), MPO (28.8 IU/mL) and GBM (94 IU/mL). Renal biopsy demonstrated numerous glomerular crescents, widespread IgG4-positive lymphoplasmacytic infiltrate and mesangial positivity for IgA. PTU was stopped and he was treated with steroids, plasma exchange and cyclophosphamide with sustained improvement in his renal function.

**Conclusions:**

This case of drug-induced AAV presented a unique and intriguing collection of serological and histological features. We propose that the PTU-induced AAV resulted in epiphenomena of anti-GBM antibody production and an IgG4-cell-rich tubulointerstitial infiltrate. It is uncertain whether the mesangial IgA deposition preceded or resulted from the AAV.

## Background

A number of disease processes can culminate in rapidly progressive glomerulonephritis (RPGN), including pauci-immune focal segmental necrotising glomerulonephritis, frequently seen with positive serum antineutrophil cytoplasmic antibodies (ANCA). Drug-induced ANCA-associated vasculitis (AAV) is well recognised and propylthiouracil (PTU) is a frequently implicated drug [1, 2]. The frequency of ANCA seropositivity in patients treated with PTU ranges from 15 to 64% in cross-sectional studies, although only a minority of these develop clinical vasculitis [1]. Antibodies against myeloperoxidase (MPO) are most frequently reported in PTU-induced AAV, with antibodies to other antigens including proteinase 3 (PR3) found less frequently [2]. While primary AAV is typically pauci-immune on renal biopsy, immune complex deposition has been reported in PTU-associated AAV [1, 3]. “Double positive” vasculitis, with anti-glomerular basement membrane (GBM) and anti-MPO seropositivity, has been previously reported in a case of PTU-associated pulmonary-renal syndrome, with histological evidence of anti-GBM disease [4].

We herein describe an unusual case of PTU-associated renal disease, with antibodies detected in the serum directed against MPO, PR3 and GBM, accompanied by histological evidence of IgA nephropathy and a tubulointerstitial infiltrate rich in IgG4-positive cells.

## Case presentation

A 51-year-old Caucasian man was referred to his local hospital with impaired renal function and haemato-proteinuria. He was an ex-smoker and had a background of Graves’ disease and asthma.

His serum creatinine at presentation was 568 μmol/L (6.4 mg/dL), compared to 116 μmol/L (1.3 mg/dL, estimated glomerular filtration rate [eGFR] 61 ml/min/1.73m^2^) 1 year prior. His CRP was 100 mg/L. Point-of-care urinalysis revealed 3+ blood and 3+ protein. Ultrasound of the renal tract was unremarkable. The patient felt well and denied any current or recent symptoms of systemic illness, specifically denying visible haematuria, dyspnoea, cough, haemoptysis, arthralgia, rash, oral ulceration or peripheral oedema. He reported no change in his urine output. Physical examination was unremarkable. He was clinically euthyroid and was normotensive. He was transferred to the regional renal unit for further investigation.

Further enquiry revealed that his Graves’ disease had been diagnosed 2 years previously and he was positive for antibodies against thyroid peroxidase at that time. He was initially treated with carbimazole but this was changed to PTU soon after due to the development of mood disturbance. He stopped this treatment 3 months prior to admission without seeking medical advice, but rapidly began to feel unwell with lethargy, weight loss, fever and worsening goitre, so propylthiouracil was re-started 2 weeks prior to presentation.

His family history revealed that his grandmother died of renal failure and his mother also suffers from kidney dysfunction. The patient was unable to provide further details regarding this. There was also a family history of autoimmune thyroid disease.

**Fig. 1:**
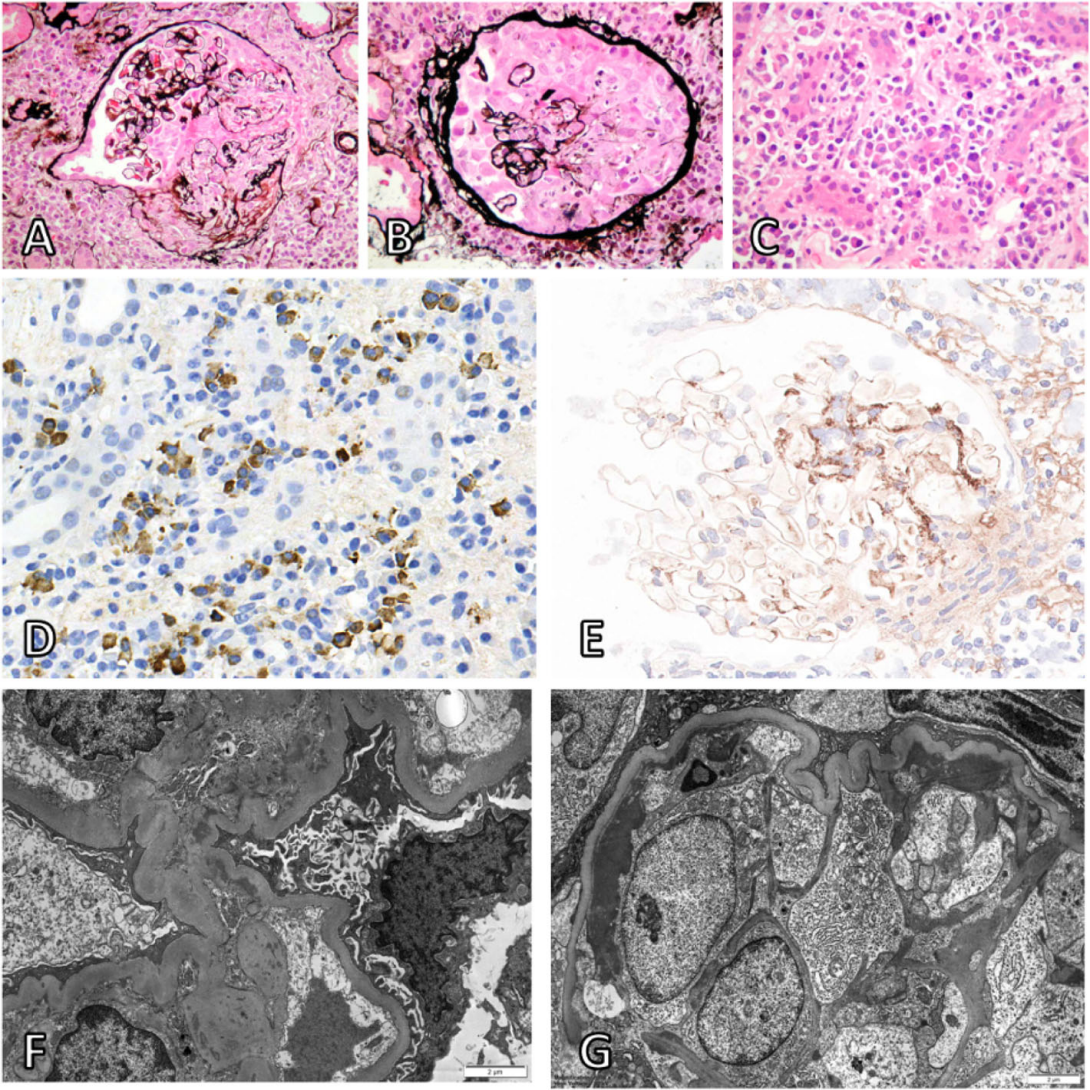
Histology from renal biopsy at 40x magnification (**a-e**). Segmental necrosis with rupture of glomerular tuft and Bowman’s capsule (**a**; Jones silver-H&E stain). Large cellular crescent (**b**; Jones silver-H&E stain). Plasma cell rich tubulointerstitial infiltrate (**c**; H&E stain). Tubulointerstitial infiltrate rich in IgG4-positive cells (**d**; IgG4 immunohistochemical staining, IgG4-positive cells stained brown). Predominantly mesangial distribution of IgA (**e**; Immunoperoxidase staining for IgA). Electron microscopy at 6000x magnification showing paramesangial electron dense deposits (**f**) and subendothelial deposits associated with endocapillary hypercellularity (**g**) typical of IgA nephropathy

A renal biopsy (Fig. 1) undertaken the day after admission demonstrated cellular crescents with little or no organisation in 50% of glomeruli, with segmental necrosis and moderate chronic damage (40% tubular atrophy), and widespread lymphoplasmacytic infiltrate, associated with tubulitis. High-dose pulses of intravenous methylprednisolone were administrated for 3 days. His propylthiouracil was stopped and low-dose carbimazole was commenced.

The day after the biopsy, autoimmune serology demonstrated positive titres for anti-GBM (94 IU/mL), p- and c-ANCA (1:80 titre), anti-PR3 (76.9 IU/mL) and anti-MPO (28.8 IU/mL). His serum creatinine was then 448 μmol/L (5.1 mg/dL). Serum protein electrophoresis showed a polyclonal hypergammaglobulinaemia and he had mildly elevated levels of immunoglobulin G (17.77 g/L) and immunoglobulin A (4.08 g/L). In view of the triple-positive immunology, histological confirmation of crescentic glomerulonephritis (GN) and severe renal dysfunction, plasma exchange was performed for eight sessions over consecutive days. At this point his anti-GBM titre had fallen to within the normal range. He also received induction therapy with intravenous cyclophosphamide and oral glucocorticoids. His creatinine improved to 399 μmol/L (4.5 mg/dL) and he was discharged from hospital, receiving two further doses of intravenous cyclophosphamide as an outpatient. Further cyclophosphamide doses were not given because he developed a lower respiratory tract infection. He did not require renal replacement therapy and did not develop evidence of pulmonary haemorrhage.

Immunofluorescence of his renal biopsy demonstrated mesangial positivity for IgA (ranging from 1+ to 3+), C3 (1+) and lambda (ranging from 0 to 2+). IgG, IgM, C1q and kappa staining were negative; specifically, there was no linear IgG staining on the glomerular basement membrane. Electron microscopy also demonstrated paramesangial electron dense deposits (Fig. 1f) and subendothelial deposits associated with endocapillary hypercellularity (Fig. 1g) typical of IgA nephropathy. In view of the lymphoplasmacytic infiltrate, IgG4 staining was subsequently undertaken. This demonstrated > 30 IgG4 positive plasma cells per high power field (Fig. 1d). Serum IgG4 concentration was not checked during the index admission but was normal on subsequent testing.

**Fig. 2:**
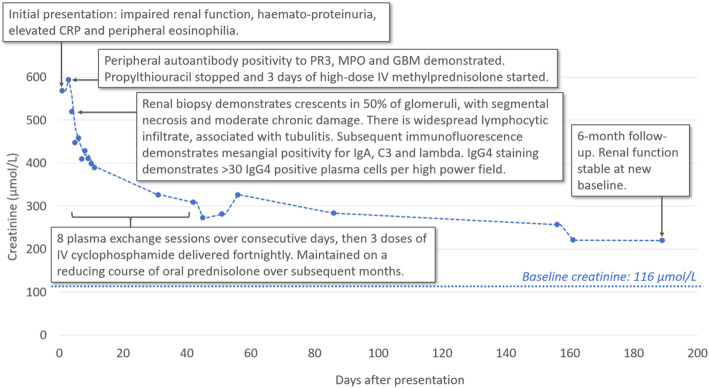
Timeline of admission and subsequent treatment overlaid on serum creatinine plot

His renal function continued to improve and stabilised with a creatinine of 254 μmol/L (2.9 mg/dL, eGFR 23 ml/min/1.73m^2^) at 10 months after index treatment (Fig. 2). His anti-GBM, IgG4 and ANCA titres remain within the normal range 3 months after completing a reducing course of prednisolone and without further immunomodulatory therapy.

## Discussion and conclusions

Drug-induced AAV is a well described phenomenon associated with a number of drugs including PTU [2]. This is an unusual case AAV presenting soon after recommencement of PTU, with serum antibodies against MPO, PR3 and GBM, accompanied by histological evidence of IgA nephropathy and an IgG4-cell-rich tubulointerstitial infiltrate.

Several mechanisms have been proposed for PTU-induced AAV. Firstly, PTU has been shown to alter the structure of MPO, which may then serve as a neoantigen. In addition, metabolites of PTU have been shown to be immunogenic, activating B cells to produce ANCA in a T-cell-dependent manner [1]. Double-positive anti-GBM and AAV is well recognized [5]. In such cases, glomerular damage caused by ANCA may expose the GBM, resulting in anti-GBM production [6]. For our patient, we propose that this was the likely mechanism of anti-GBM seropositivity, secondary to AAV caused by anti-MPO and/or anti-PR3. Unlike a previously reported case of PTU-associated double positive anti-GBM and AAV [4], it is unlikely that the anti-GBM antibodies contributed to our patient’s renal impairment, since his clinical presentation was relatively mild with no pulmonary haemorrhage, and he did not have histological evidence of anti-GBM disease, specifically a lack of linear IgG deposition on the glomerular basement membrane. The good recovery of renal function in response to immunotherapy seen in our patient is also more consistent with AAV.

Interestingly, our patient also demonstrated dense lymphoplasmacytic infiltration with IgG4-positive plasma cells. Again, it is uncertain whether this contributed significantly to his clinical presentation. IgG4-related disease (IgG4-RD) is a chronic fibro-inflammatory condition with a poorly understood immunopathogenesis. Initially described in autoimmune pancreatitis, the features of tumour-like lesions, dense lymphoplasmacytic infiltrates and abundant IgG4-bearing plasma cells are common to all affected tissues [7]. To our knowledge, no association between drugs and IgG4 disease has been described to date. In our patient, the presence of abundant IgG4-positive cells in the tubulointerstitial infiltrate, accompanied by acute kidney injury (AKI) and an active urinary sediment, fulfils the diagnostic criteria for ‘probable IgG4-related kidney disease’ (IgG4-RKD) [8]. However, the presence of an IgG4-rich infiltrate is not specific to IgG4-RKD and has been previously reported in cases of AAV [9]. In common with our patient, aside from the IgG4-positive infiltrate, these cases had no clinical or histological evidence of IgG4-RD. Conversely, cases of biopsy-proven concomitant IgG4-RKD with both AAV and anti-GBM disease have been reported [10, 11]. Graves’ disease with elevated IgG4 has been analysed in several studies, though no patients have previously been reported to have extra-thyroid involvement [12]. We advised our patient to undergo cross-sectional imaging to check for evidence of IgG4-RD affecting other organs, but he declined further investigation.

Immunofluorescence in our patient also demonstrated mesangial positivity for IgA. It is conceivable that the autoimmune cascade that promoted the AAV and anti-GBM antibodies in our patient may have promoted T-cell overactivation, leading to increased production of cytokines such as ‘a proliferation-inducing ligand’ (APRIL), which are known to promote B cell class switch to IgA1-producing plasma cells, and contributed to the IgA mesangial positivity [13]. However, it is also possible that the patient had pre-existing subclinical IgA nephropathy which was unmasked by the additional renal insult. An eGFR at the lower limit of normal for age 1 year prior to index presentation is suggestive of pre-existing renal pathology, but the lack of previous biopsy samples or urinalysis will leave this question unanswered.

We postulate that this presentation was induced by PTU, since it had been recently restarted. Our patient had previously been treated briefly with carbimazole, which has also been implicated in drug-induced AAV [2], but our patient’s sustained improvement in renal function and resolution of autoimmune seropositivity despite re-commencement of carbimazole makes it an unlikely culprit. PTU-associated AAV tends to be less severe than primary AAV, which is in line with the relatively mild clinical presentation in our patient [3]. The mechanism of induction of AAV by PTU is a subject of ongoing debate. One theory is that PTU may alter the conformation of neutrophil extracellular traps (NETs), triggering an autoimmune response. Indeed, administration of NETs exposed to PTU triggered MPO AAV in a rat model [14].

The patient was treated as per anti-GBM/AAV/Crescentic IgAN KDIGO protocols, with good response despite incomplete cyclophosphamide therapy due to intercurrent infection. The use of plasmapheresis in addition to corticosteroids and cyclophosphamide is recommended for both anti-GBM disease and anti-GBM/AAV overlap [15]. Drug-induced AAV is, in many ways, an atypical form of vasculitis and optimal therapy in the presence of a specific aetiological agent, other than withdrawal of the postulated trigger, has not been established. The need for cytotoxic induction therapy beyond plasma exchange and high-dose steroids once the trigger has been removed is not clear. In this case, despite ‘incomplete’ induction for AAV he rapidly achieved quiescence without evidence of relapse despite a lack of maintenance therapy.

In conclusion, we have described an interesting case of PTU-induced AAV with serum antibodies against MPO, PR3 and GBM, accompanied by histological evidence of IgA nephropathy and an IgG4-cell-rich tubulointerstitial infiltrate. We propose that both the anti-GBM antibodies and the IgG4 infiltrate are likely to be epiphenomena of AAV in this case and did not play a significant role in the pathogenesis of his AKI. It is also uncertain whether mesangial IgA deposition preceded or resulted from the AAV. Despite significant disease activity at presentation, the patient had no extrarenal manifestations and showed reasonable recovery of renal function with cessation of PTU, plasmapheresis, corticosteroid and cyclophosphamide treatment.

## Data Availability

Not applicable.

## Abbreviations

AAV: ANCA-associated vasculitis
AKI: Acute kidney injury
ANCA: Antineutrophil cytoplasmic antibodies
APRIL: A proliferation-inducing ligand
GBM: Glomerular basement membrane
GN: Glomerulonephritis
IgG4-RD: IgG4-related disease
IgG4-RKD: IgG4-related kidney disease
MPO: Myeloperoxidase
NETs: Neutrophil extracellular traps
PR3: Proteinase 3
PTU: Propylthiouracil
RPGN: Rapidly progressive glomerulonephritis
